# Supplementary material for: LITESEC-T3SS - Light-controlled protein delivery into eukaryotic cells with high spatial and temporal resolution
Source: Nat Commun. 2020 May 13;11:2381. doi: 10.1038/s41467-020-16169-w (PMC7221075; doi:10.1038/s41467-020-16169-w)
Supplement: Supplementary file 1 — Supplementary Information [file 41467_2020_16169_MOESM1_ESM.pdf]

Supplementary Information

LITESEC-T3SS - Light-controlled protein delivery into eukaryotic cells with high spatial and temporal resolution

Lindner *et al.*

Supplementary Discussion

Kinetics of LITESEC activation and deactivation

Both reaction time and recovery dynamics of the sequestration systems are crucial for their applicability to control the function of the T3SS. Fast reaction times to blue light increase the temporal precision of T3SS activation/deactivation, whereas the recovery times influence the duration of the effect on secretion after illumination. Very fast recovery means that the system has to be continuously illuminated for a sustained effect on secretion, while very slow recovery leads to long-term activation/deactivation that is difficult to revert, and renders handling of the cultures difficult due to possible long-term effects of illumination prior to the actual experiment. In time-course experiments we could show that in the iLID-based protein sequestration system, unbinding of the bait in the light state was almost immediate, and that recovery in the dark occurred within few minutes (Fig. 2), in line with data from eukaryotic systems[1]. In the resulting LITESEC-supp system, both activation and deactivation of type III secretion occur relatively quickly, within the first minutes (Supplementary Fig. 8), which is in the range of the measured turnover of SctQ at the injectisome (half-time of about 70 s under secreting conditions[2]). This suggests that the release and rebinding of the bait protein occurs faster or in a similar time range, consistent with the microscopy results (Fig. 2). For the LITESEC-act system, we detected a slower activation and deactivation of protein secretion (Supplementary Fig. 8). Nevertheless, induction of protein secretion by blue light occurs within minutes (Supplementary Fig. 8). Importantly, in the absence of further illumination, protein secretion is stopped within minutes, which greatly limits unwanted unspecific activation. Long-term activation can be achieved by either constant low-intensity blue light illumination, or short light pulses every few minutes. Ambient laboratory light did not inhibit the LITESEC-supp2 strains, but lead to an intermediary activation of LITESEC-act3 (Supplementary Fig. 5).

Current and possible future applications of the LITESEC system

A main application of the LITESEC system is the temporally and spatially controlled translocation of proteins into cultured eukaryotic cells (Fig. 8). Cell cultures play an important role in research, development and, increasingly, healthcare. Often, specific proteins need to be expressed in all or a subset of the cultured cells at a given time point. At the moment, this is mainly achieved by inducing expression of the target protein within the host cells. This method requires prior transfection of the host cells with the target gene or time-consuming creation of stable transgenic cell lines. Induction of expression itself is relatively slow, and difficult to apply to a certain subset of cells. Our method allows to translocate proteins into unmodified host cells with high specificity. Bacteria that lack their native virulence effectors (such as the *Y. enterocolitica* strain used in this study), but express one or more cargo proteins with a short secretion signal, are brought into contact with host cells. The chosen subset of host cells are then subjected to dark or blue light conditions (which does not influence bacteria or host cells at the used intensity), which temporarily induces translocation of the cargo into the host cells within short time. An additional advantage of the LITESEC method is that it directly translocates proteins into

the host cell, rather than inducing the transcription of mRNA, as is the case in the current inducible transfection systems. The amount of translocated protein can be regulated by the duration of illumination/darkness, and the multiplicity of infection (ratio of bacteria / host cells)[3].

A potential, relatively straightforward extension of our work would allow the specific protein delivery into diseased cells, such as cancer cells, within biological tissues. The T3SS has been used to treat cancer cells *in vitro*, e.g. by translocating angiogenic inhibitors[4], but again, the promiscuity of the T3SS and the resulting unspecific translocation at non-target sites represent a major obstacle in the further development of T3SS-based methods for clinical applications[5]. Most current approaches rely on localized injection of bacteria or the natural tropism of bacteria to tumorous tissue. However, bacteria applied with these methods are not restricted to the target tissue, and unspecific activation presents a problem, especially for potentially powerful applications such as the delivery of pro-apoptotic proteins. By using light to specifically activate the modified T3SS in bacteria at a site of choice, delivery of effector proteins could be temporarily induced at a specific time and place. This method would reduce unspecific activation and side effects, allowing a highly controlled targeting of host cells. Bacteria could be applied to the patient (exploiting the natural tropism of bacteria for tumor tissue to achieve local enrichment in the case of cancer), where injection of the effector protein would be triggered *in situ* with high spatial and temporal precision using light delivered with the help of endoscopes and minimally-invasive surgery techniques. As the blue light used to control the current LITESEC systems does not penetrate tissue efficiently, activation by red or far-red light would be advantageous. Several such red-light systems have been characterized[6–8]; however, all of these systems require cofactors not usually present in bacteria.

Supplementary Figures

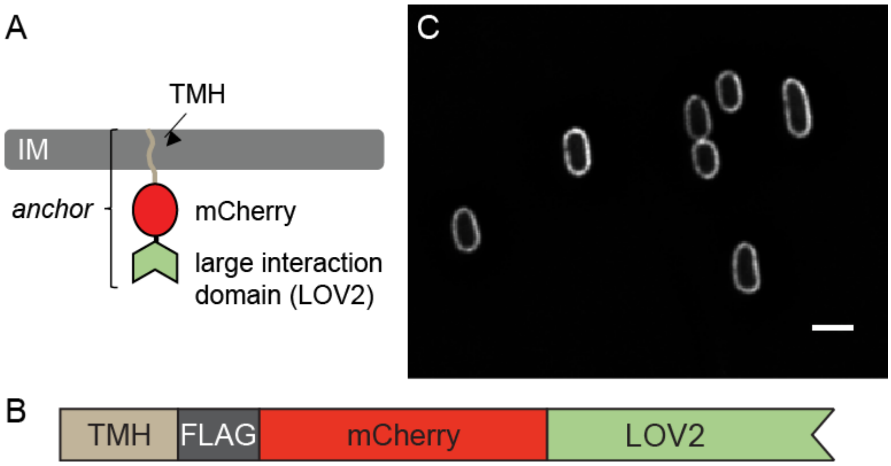

Supplementary Fig. 1: Membrane localization of the LOV anchor protein in *Y. enterocolitica*

(**A**, **B**) Schematic depiction of the mCherry-labeled version of the membrane anchor protein for the LOVTRAP-based membrane sequestration system, TMH-FLAG-mCherry-LOV2. (**C**) Localization of TMH-FLAG-mCherry-LOV2 determined by widefield fluorescence microscopy. Scale bar, 2 µm.

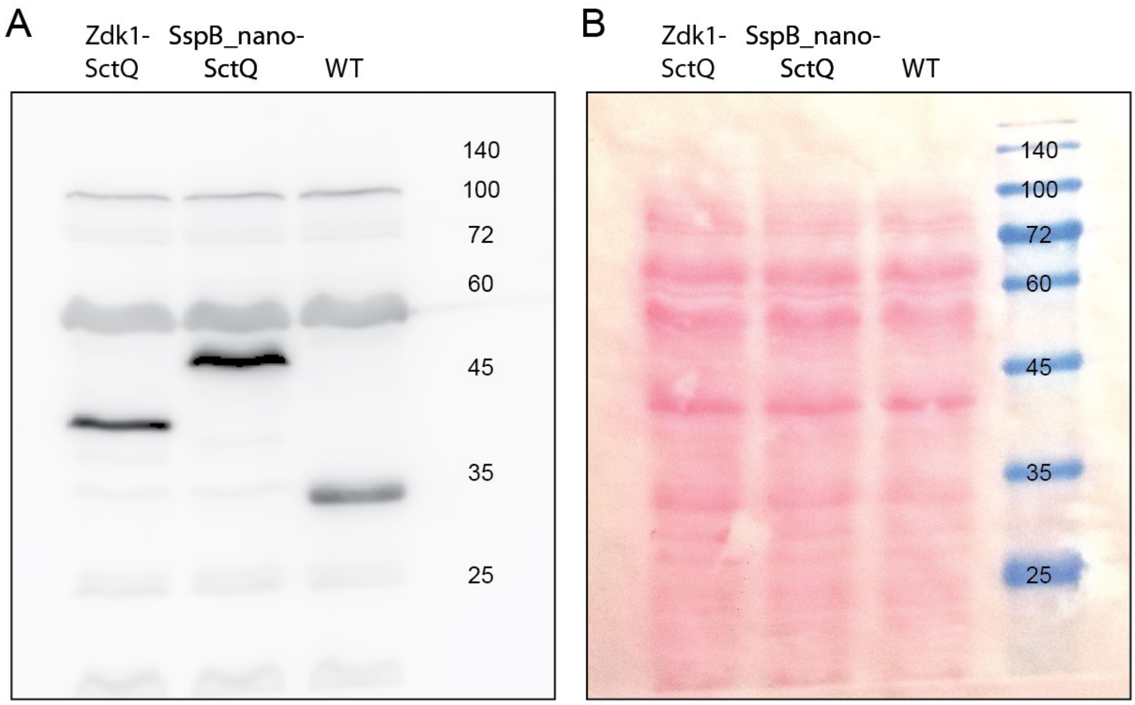

Supplementary Fig. 2 – The used fusion proteins are stable.

(**A**) Western blot anti-SctQ of the used bait-SctQ fusion proteins, expressed from the native genetic locus on the virulence plasmid. Detected proteins and expected sizes: Zdk1-SctQ, 40.8 kDa; SspB_Nano-SctQ, 46.7 kDa; WT SctQ, 34.4 kDa. Right side, molecular weight marker (weight in kDa). (**B**) Ponceau staining of the membrane used for (A). Source data are provided as a Source Data File.

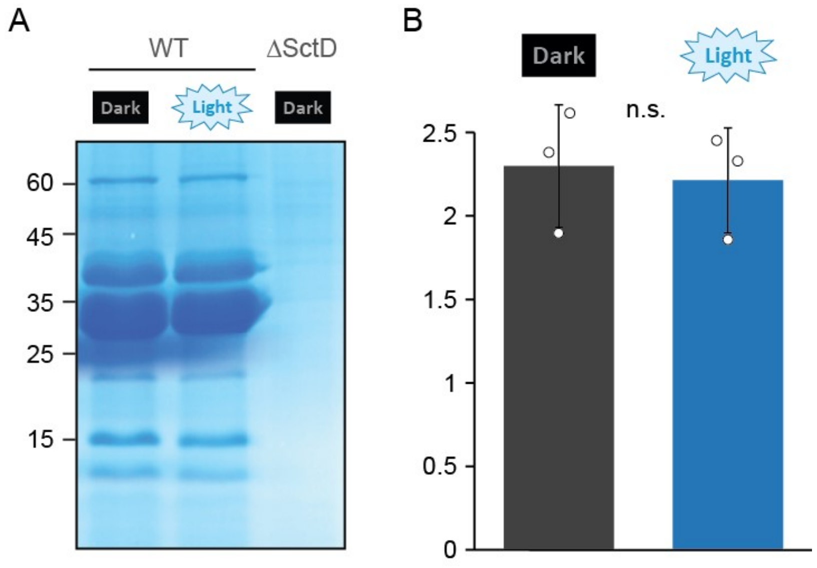

Supplementary Fig. 3: Blue light illumination in the used intensity does not significantly influence growth, division, or T3SS activity of *Y. enterocolitica*

(**A**) *In vitro* secretion assay showing export of T3SS substrates in the indicated strains (WT, wild-type; ΔSctD, T3SS-negative control) in dark or light (constant low-level illumination of ~ 1 mW cm^{-2} at λ=488 nm) conditions, as indicated. Proteins secreted by $3*10^8$ bacteria during a 180 min incubation period were precipitated and analyzed by SDS-PAGE. Left side, molecular weight in kDa. (**B**) Average optical density at 600 nm of wild-type cultures in secreting conditions after 180 min in dark conditions (grey, left) or light conditions (blue, right) as used in the optogenetics experiments. N=3 optical density values from independent experiments; single data points indicated by circles. Error bars display standard deviation; n.s., no statistically significant difference (p=0.77 in a two-tailed homoscedastic t-test). Source data are provided as a Source Data File.

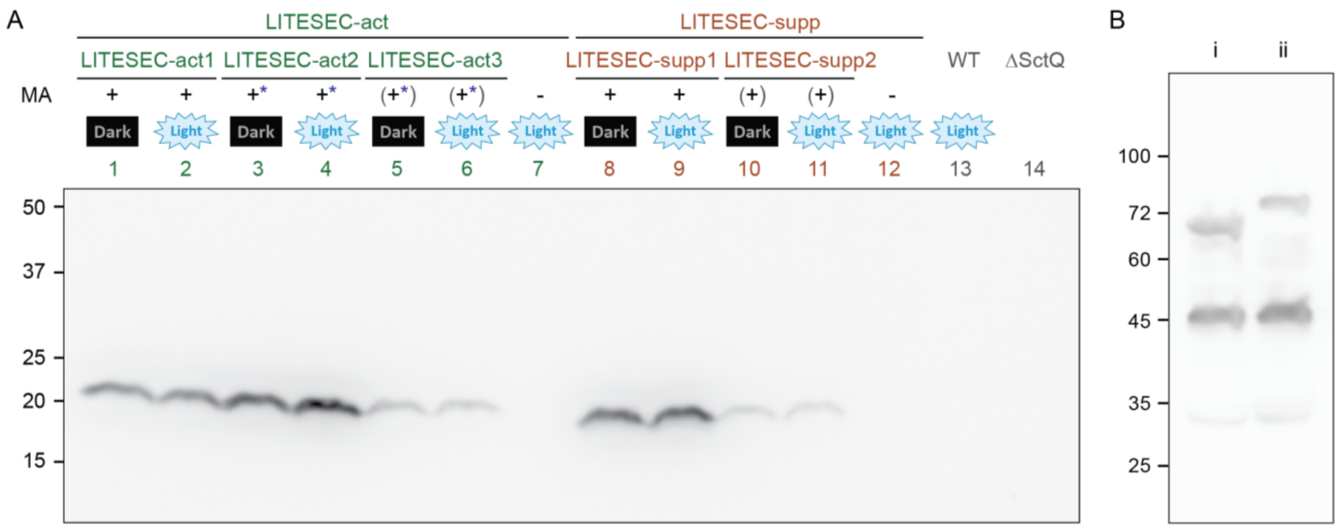

Supplementary Fig. 4: Expression levels of membrane anchor proteins in the different LITESEC variant strains

(**A**) Western blot anti-FLAG of total cellular protein from $2*10^8$ bacteria in the indicated strains (corresponding to Fig. 4). Left, molecular weight marker in kDa. Expected protein sizes: 20.9 kDa for LITESEC-act strains (TMH-FLAG-LOV2 / TMH-FLAG-LOV2$_{V416L}$), 21.6 kDa for LITESEC-supp strains (TMH-FLAG-iLID). MA, expression level of membrane anchor; +, high expression level; (+), low expression level; -, no expression. *, V416L anchor mutant. (**B**) Western blot anti-mCherry of mCherry-labeled anchor and bait combinations of both LITESEC systems. Detected proteins and expected sizes: i, Zdk1-mCherry-SctQ and TMH-FLAG-mCherry-LOV2, 67,8 and 47,6 kDa; ii, SspB_Nano-mCherry-SctQ and TMH-FLAG-mCherry-iLID, 73,7 kDa and 48,7 kDa. Left, molecular weight marker in kDa. N=3.

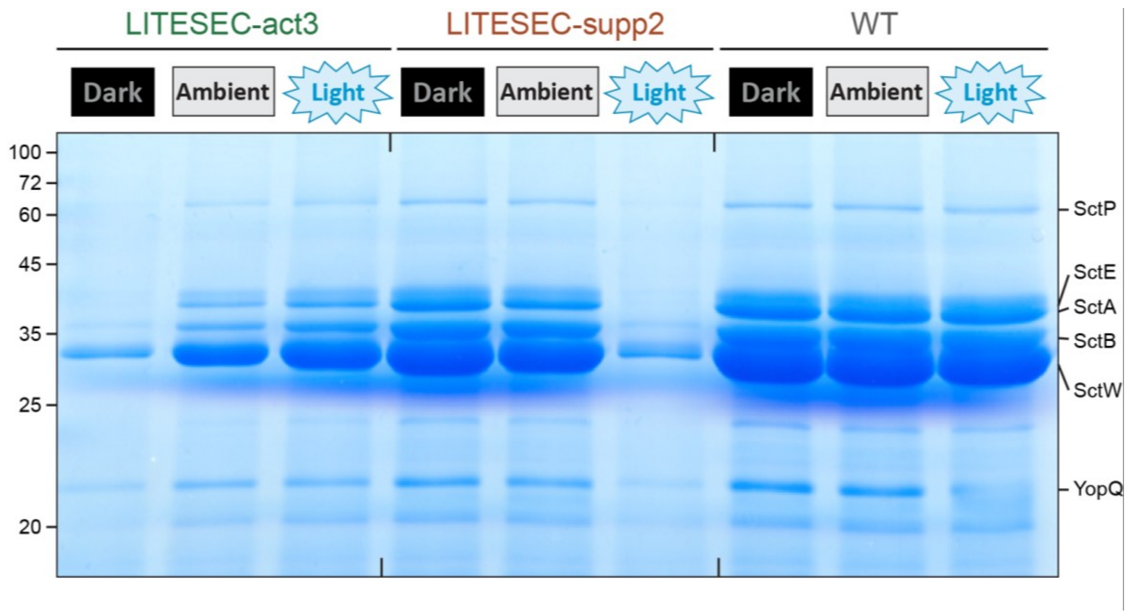

Supplementary Fig. 5: Influence of ambient light on LITESEC secretion activity

In vitro secretion assay showing light-dependent export of native T3SS substrates (indicated on the right) in the listed strains, incubated under defined dark or light conditons (see material and methods for details), as well as ambient laboratory light. Proteins secreted by $3*10^8$ bacteria during a 180 min incubation period were precipitated and analyzed by SDS-PAGE. Left side, molecular weight in kDa.

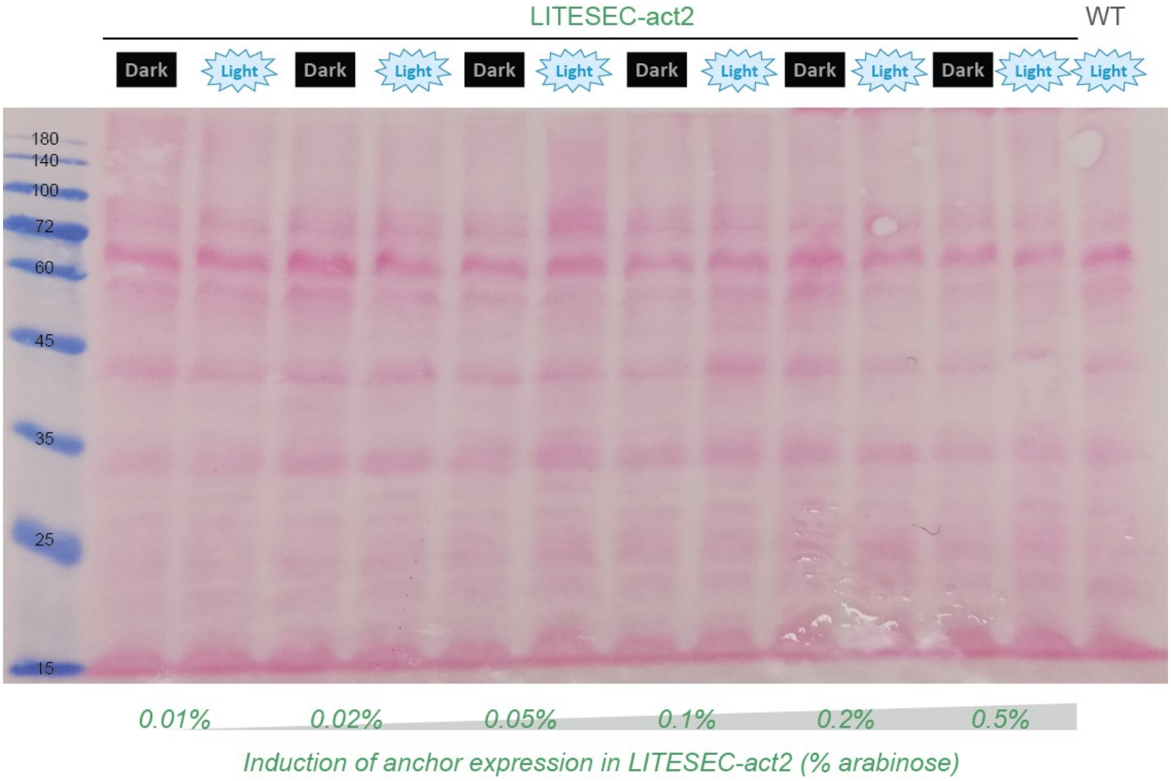

Supplementary Fig. 6: Loading control for the analysis of different induction levels of LITESEC-act2 membrane anchor.

Ponceau staining of a Western blot of total cellular protein of $2*10^8$ bacteria of the LITESEC-act2 and corresponding wild-type (WT) strain at the indicated induction levels and conditions, corresponding to Figure 5C. Left side, molecular weight in kDa.

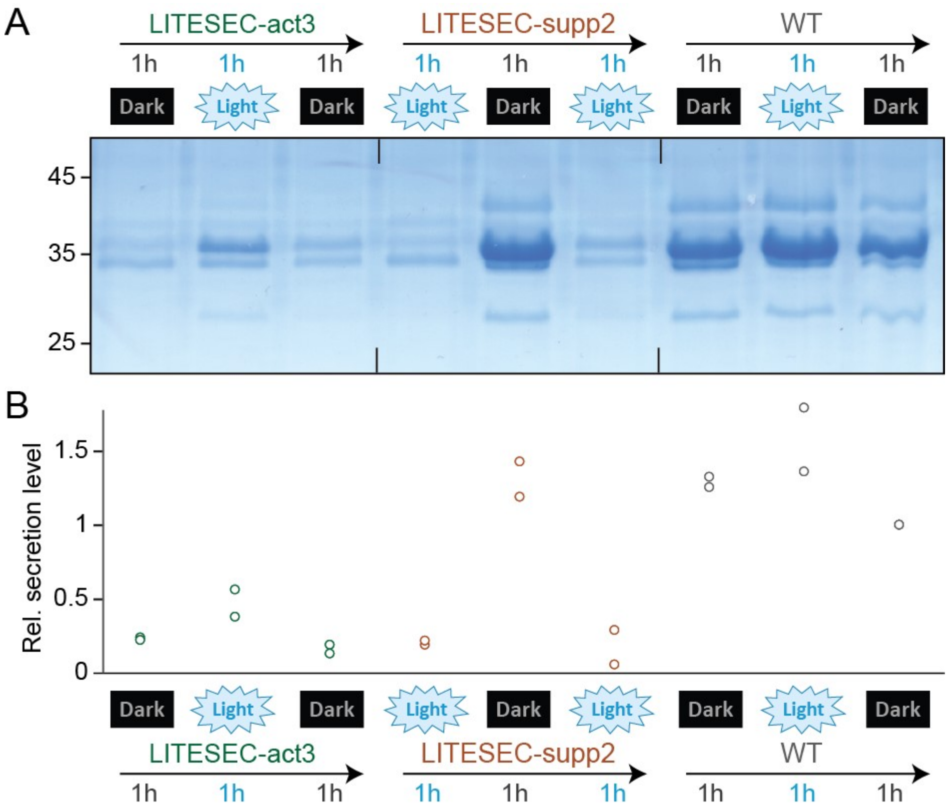

Supplementary Fig. 7: Control of secretion of native T3SS substrates over time.

(**A**) Export of native T3SS substrates in the indicated strains. Secretion-competent bacteria were subsequently incubated under inactivating, activating and inactivating light conditions for 60 min each, as in Fig. 7 and as indicated, and the supernatant of $3*10^8$ bacteria was analyzed. Left side, molecular weight in kDa. (**B**) Quantification of the relative export efficiency (normalized to the wild-type level in the third incubation period) of the strains; n=2, single data points shown. Source data are provided as a Source Data File.

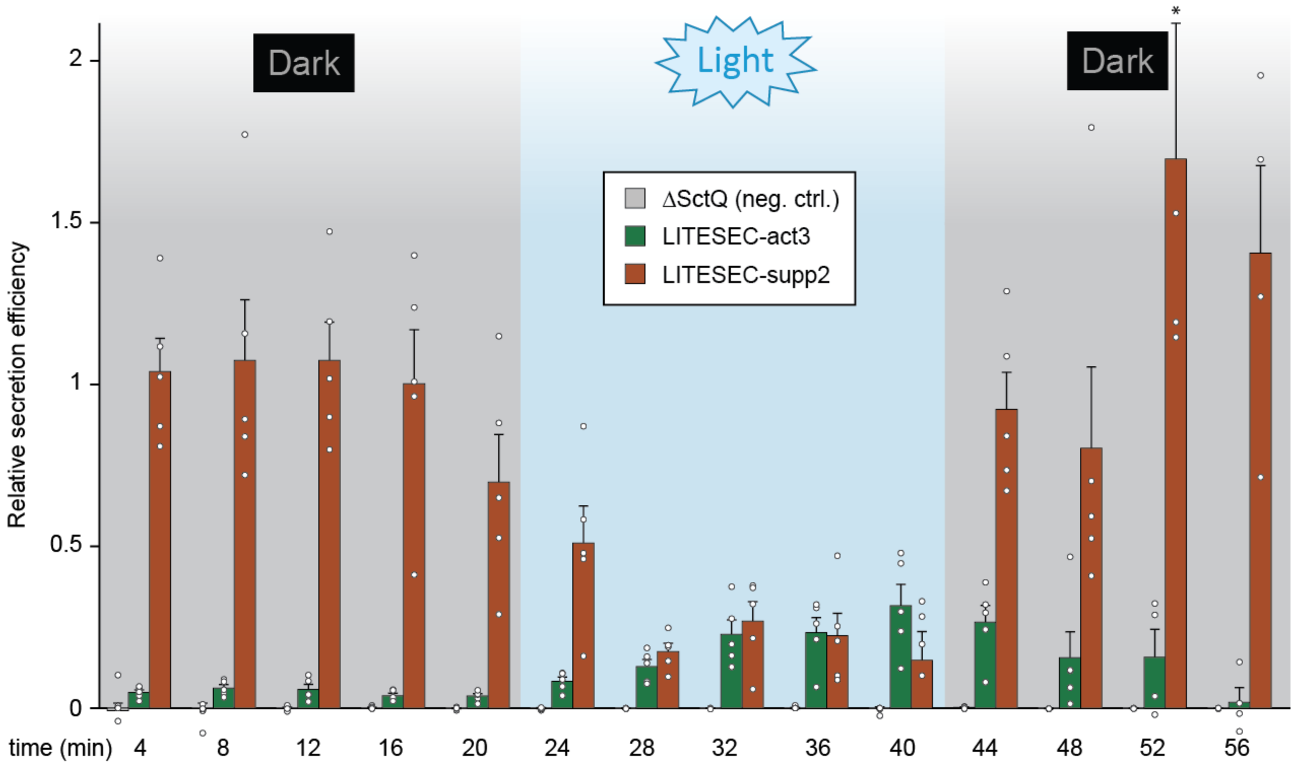

Supplementary Fig. 8: Determination of switching kinetics in the LITESEC strains

Secretion-competent bacteria were incubated under the indicated conditions. Every four minutes, samples were removed, in which secretion was stopped by addition of 10 mM $CaCl_2$, and bacteria were harvested by centrifugation. Secretion of $YopE_{1\text{-}53}$-NanoLuc-FLAG was quantified by a luciferase-based luminescence assay in a plate reader. The increase in secretion in comparison to the previous time point was determined and normalized for the secretion of a wild-type strain. Values for the negative control are too small to see in some cases. N=5; single data points displayed as circles (*, one data point off chart at *y*=2.93), error bars denote the standard error of the mean. Source data are provided as a Source Data File.

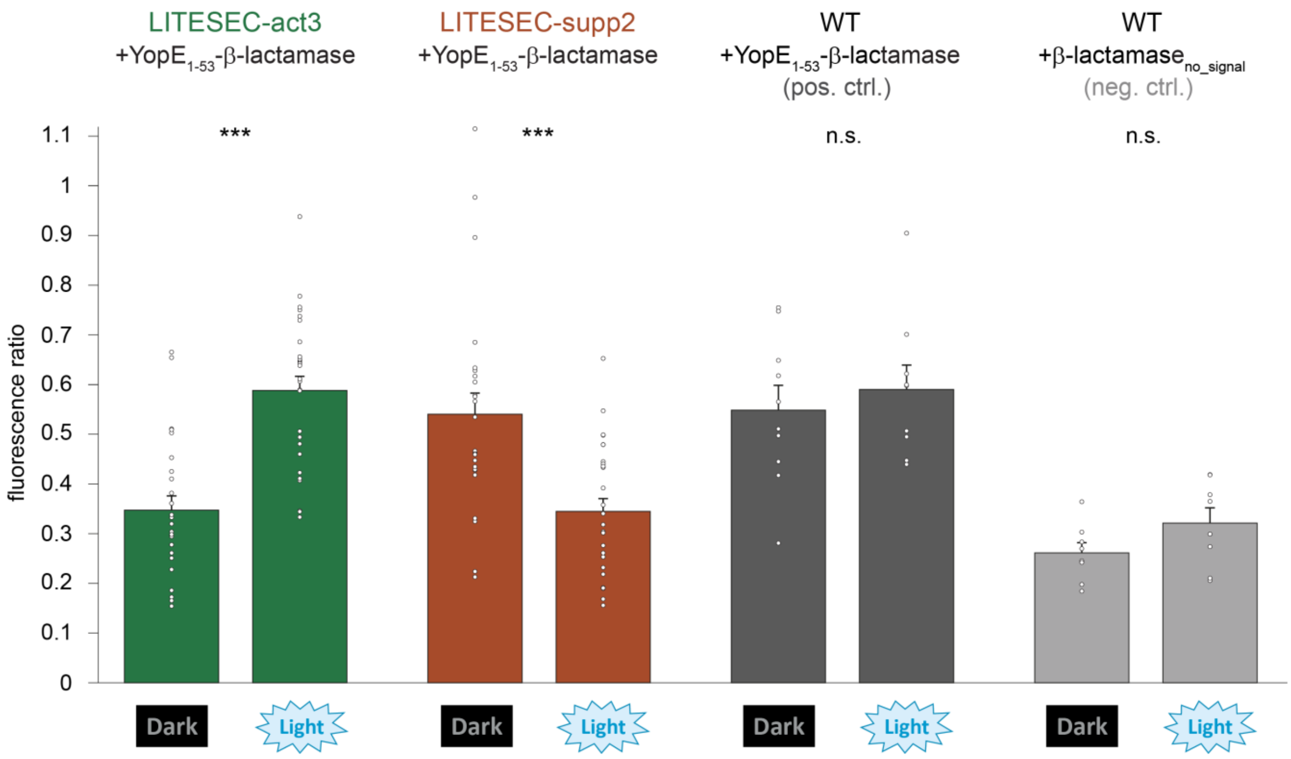

Supplementary Fig. 9: Additional quantification of light-dependent translocation efficiency

Quantification of the fluorescence ratio of CCF2 donor fluorescence (indicative of β-lactamase translocation) and FRET fluorescence for the infection experiment shown in Fig. 8A. 2343/2423/2226/2694 cells from 26/28/25/27 fields of view from 3 independent experiments were analyzed for the LITESEC strains under the given conditions from left to right (809/671/995/823 cells from 8/8/10/9 fields of view from 3 independent experiments for the controls). Fluorescence ratios per single fields of view are displayed as circles; error bars display the standard error of the mean. ***, $p<0.001$ in a two-tailed homoscedastic t-test; n.s., difference not statistically significant (exact values from left to right, $6*10^{-7}/2*10^{-4}/0.54/0.13$). Source data are provided as a Source Data File.

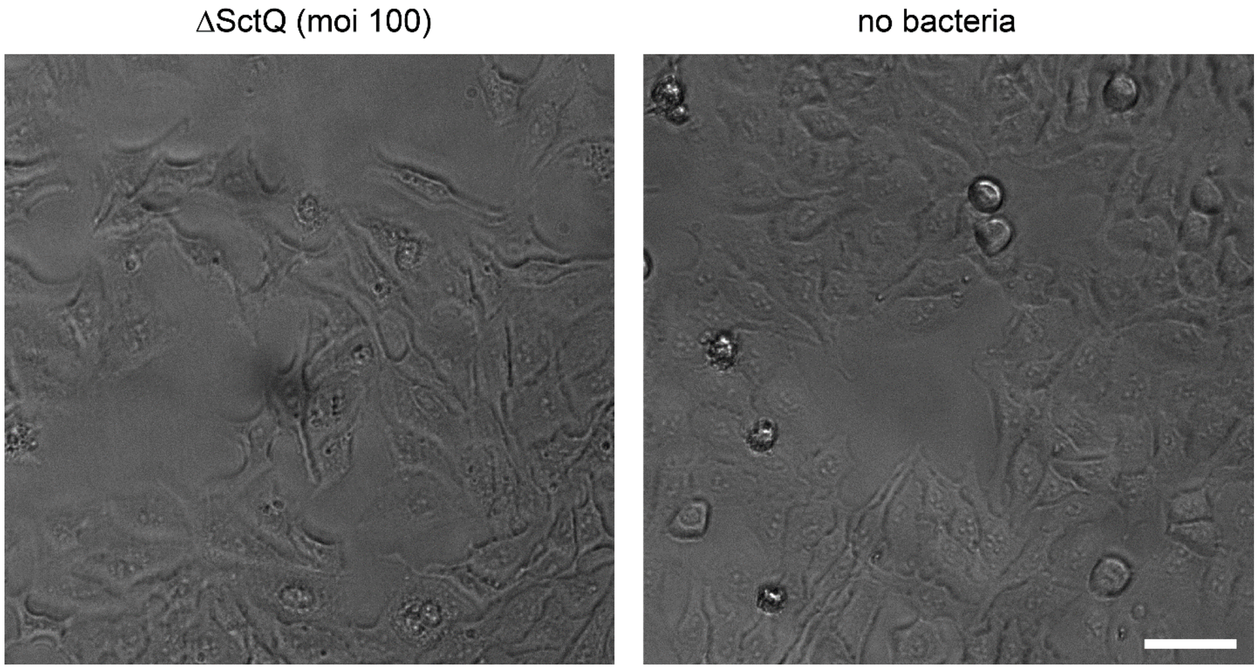

Supplementary Fig. 10: Host cells show no visible reaction to T3SS-inactive *Y. enterocolitica*.

Left, HEp-2 cells were infected with ΔSctQ bacteria for 1 h at a multiplicity of infection (moi) of 100, as in Fig. 8. After removal of the bacteria, cells were incubated in medium containing gentamicin for further 17 h to detect possible long-term effects of the presence and contact of bacteria. Right, control incubated under the same conditions without bacteria. Scale bar, 50 µm.

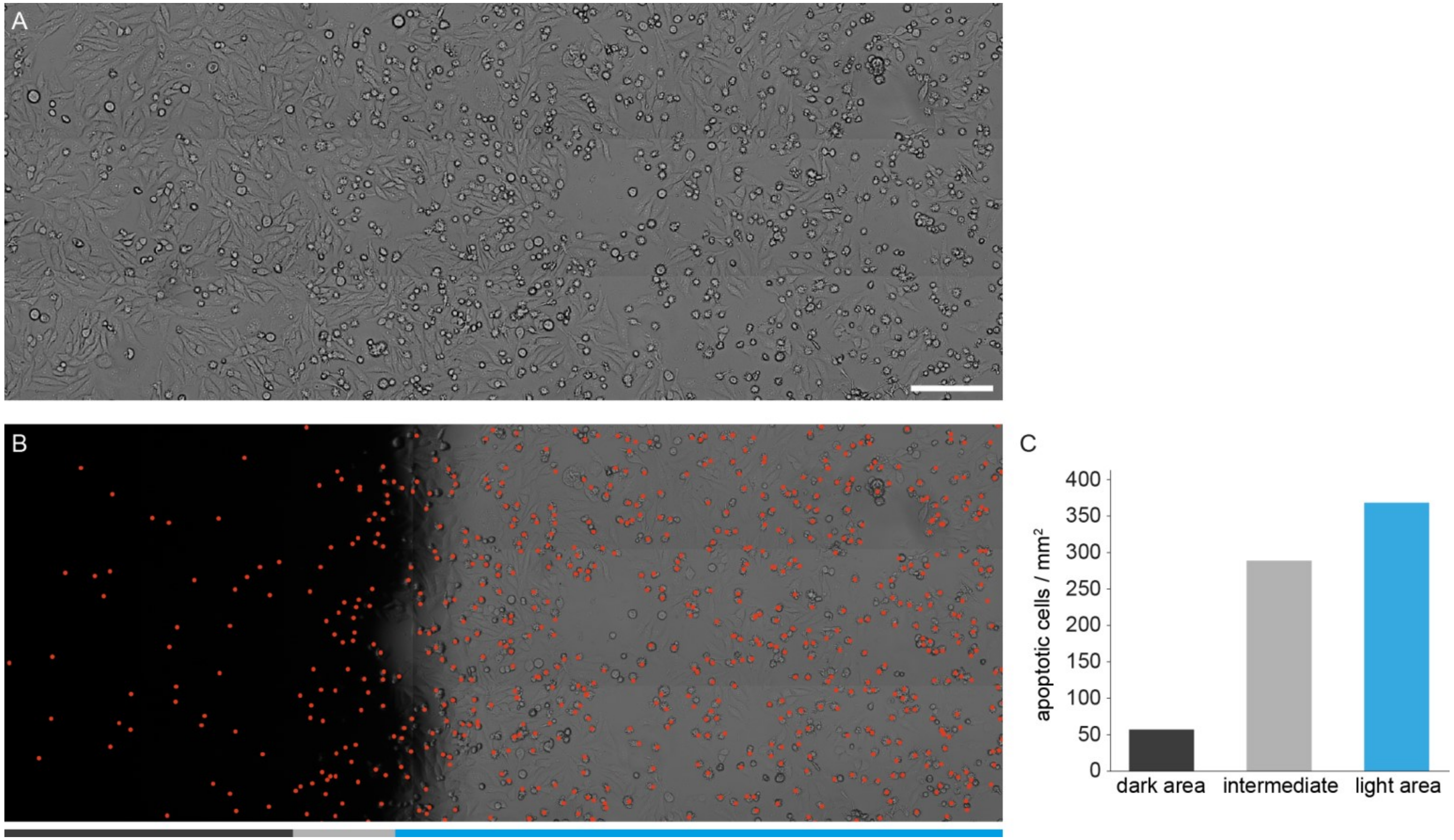

Supplementary Fig. 11: Spatial resolution of apoptosis induction by LITESEC-act3 bacteria

HEp-2 cells were infected with LITESEC-act3 bacteria expressing $YopE_{1\text{-}138}$-tBID, as in Fig. 8C-E, under light conditions. In a part of the well, light was blocked by an intransparent plastic inset. After infection, the region around the edge of the plastic sheet was imaged with and without the inset and apoptotic cells were determined by shape. (**A**) Light micrograph in the absence of the inset. Images from three adjacent rows of eight neighboring fields of view were taken with a 20x objective and combined using softWoRx 7.0.0. Scale bar, 0.2 mm. (**B**) Light micrograph of the same area in presence of the inset, showing the approximate area of illumination during infection, as well as the localization of apototic cells determined in (A) (red points). Bottom, areas assigned as dark (dark grey bar), intermediate (0.25 mm wide adjacent area, light grey bar) and light (blue bar). (**C**) Density of apoptotic cells per mm^2 in each of the areas determined in (B). Source data are provided as a Source Data File.

Supplementary Tables

Name	Genotype	Strain background	Comments* / Reference
**dHOPEMTasd**	*pYV40 $yopO_{\Delta 2\text{-}427}$ $yopE_{21}$ $yopH_{\Delta 1\text{-}352}$ $yopM_{23}$ $yopP_{23}$ $yopT_{135}$ Δasd*		Ref. [9]
**AD4324**	*mCherry-sctQ*	dHOPEMTasd	Ref. [2]
**AD4419**	*ΔsctQ*	dHOPEMTasd	Ref. [2]
**ADMT4521**	*mCherry-sctL*	dHOPEMTasd	Ref. [10]
**FL4002**	*Zdk1-mCherry-sctQ*	dHOPEMTasd	pFL115 x dHOPEMTasd
**FL4003**	*Zdk1-SctQ, mCherry-sctL*	dHOPEMTasd	pAD612 x ADTM4521
**FL4004**	*SspB_Nano-mCherry-sctQ*	dHOPEMTasd	pFL117 x dHOPEMTasd
**FL4005**	*SspB_Nano-sctQ, mCherry-sctL*	dHOPEMTasd	pFL118 x ADTM4521

Supplementary Table 1: *Yersinia enterocolitica* strains used in this study

* x = homologous recombination between mutator-plasmid and host-strain, recombination leads to an allelic exchange of the native gene and the mutated gene[11].

**Name** (Reference)	**Genotype**	**Primers for amplification**	**Template for Insert**	**System**
**pFL100** (this work)	*pBAD::TMH-FLAG-(L1)-mCherry-(L2)-LOV2*	AD638/704/705	p81041*	LOV
**pFL104** (this work)	*pACYC184::Zdk1-(L4)-mCherry*	AD638/699/ 721/722	p81010*/ pAD304	LOV
**pFL107** (this work)	*pBAD::TMH-FLAG-(L1)-mCherry-(L3)-iLID*	AD638/706/707/ 732/733	p60408*/ pAD304	iLID
**pFL108** (this work)	*pBAD::TMH-FLAG-(L1)-iLID*	AD638/733/734	p60408*	iLID
**pFL109** (this work)	*pACYC184::SspB_Nano-(L4)-mCherry*	AD721/722/ 735/736	p60409*/ pAD304	iLID
**pFL111** (this work)	*pBAD::Zdk1-(L4)-mCherry*	AD759/768	pFL104	LOV
**pFL113** (this work)	*pBAD::SspB_Nano-(L4)-mCherry*	AD762/768	pFL109	iLID
**pFL114** (this work)	*pBAD::SspB_Nano*	AD762/769	pFL109	iLID
**pFL115** (this work)	*pKNG101::Zdk1-(L4)-mCherry-sctQ*	**	pFL111	LOV
**pFL117** (this work)	*pKNG101::SspB_Nano-(L4)-mCherry-sctQ*	**	pFL113	iLID
**pFL118** (this work)	*pKNG101::SspB_Nano-sctQ*	**	pFL114	iLID
**pFL126** (this work)	*pACYC184::TMH-FLAG-(L1)-*$LOV2_{V416L}$	AD921/903	pAD610	LOV
**pFL127** (this work)	*pACYC184::TMH-FLAG-(L1)-iLID*	AD921/904	pFL108	iLID
**pFL133** (this work)	*pBAD::*$YopE_{1-53}$*-Nanoluc-FLAG*		pAD681	
**pAD304** (ref. 2)	*pUC19-mCherry*			
**pAD608** (this work)	*pBAD::TMH-FLAG-(L1)-LOV2*	AD638/639/640	p81041*	LOV
**pAD610** (this work)	*pBAD::TMH-FLAG-(L1)-*$LOV2_{V416L}$			LOV
**pAD612** (this work)	*pKNG101::Zdk1-sctQ*	**	pAD611	LOV
**pAD627** (ref. 12)	*pACYC184::*β*-lactamase*			
**pAD681** (this work)	*pBAD::*$yopH_{1-17}$*-NanoLuc-FLAG*	AD933/934	pNL1.1 (Promega)	
**pBMD028** (this work)	*pBAD::*$yopE_{1-53}$	AD894/895	pYV(MRS40)	
**pBMD040** (this work)	*pBAD::*$yopE_{1-53}$*-*β*-lactamase*	**	pAD627/ pBMD028	
**pSi_87** (ref. 3)	*pBAD::sycE-*$yopE_{1-138}$*-tBID*			

Supplementary Table 2: Plasmids used in this study

Plasmids with corresponding properties that were designed and/or used in this work. Anchor proteins were targeted to the bacterial IM by addition of an optimized TMH based on the N-terminal TMH of the *Escherichia coli* TatA protein[13], an integral component of the Tat export system[14]. A high expression ratio of the anchor to bait protein was reported to be a prerequisite for complete binding of the bait to the

anchor[15]. We therefore expressed the membrane anchor constructs from the inducible medium-high expression vector pBAD-His/B, and the cytosolic bait fusions from a compatible constitutive low expression vector, pACYC184. * Addgene code; ** Restriction and ligation only; L1 – GAGG linker; L2 – GSGS linker;L3 – GAGGGAGG linker; L4 – GGSGGSGG linker.

Primer	Sequence from 5‘ to 3‘	Reference
AD638	GGTCTCCC**ATG**GGTGGTATCAGTATTTGGCAGTTATTGATTATTGCCGTCATCGTTGTACTGCTTGTCCTATTTGGCACCAAAAAGCTCGGCTCCGACTACAAGGACGACGATGATAAGGGTGGAGCAGGT	this work
AD639	GACTACAAGGACGACGATGATAAGGGTGGAGCAGGTGGATCCTTGGCTACTACACTTGA	this work
AD640	GACTGAATTCGCAAGCTT*TTA*AAGTTCTTTTG	this work
AD698	GACTGGATCCT*TGACTGA*ATGGTGGATAACAAATTCAATAAAGAAAAGA	this work
AD699	ACCACCAGAGCCGCCCGACCCACCAGAACCACCTTTTGGGGCCT	this work
AD700	GGTGGGTCGGGCGGCTCTGGTGGTGGTGCTGGCGTGAGCAAG	this work
AD701	GATCGTCGACTTACTTGTACAGCTCGTCCATGC	this work
AD704	GACGATGATAAGGGTGGAGCAGGTGTGAGCAAGGGCGAGGAG	this work
AD705	GACTGAATTCGCAAGCTTTTAAAGTTCTTTTG	this work
AD706	GACGATGATAAGGGTGGAGCAGGTGTGAGCAAGGGCGAGGAG	this work
AD707	TCCACCTGCTCCACCACCAGCGCCCTTGTACAG	this work
AD721	GGTGGGTCGGGCGGCTCTGGTGGTGTGAGCAAGGGCGAGGAG	this work
AD722	GATCGTCGACTTACTTGTACAGCTCGTCCATGC	this work
AD732	GGCGCTGGTGGTGGAGCAGGTGGAGGATCCGGGGAGTTTCTGG	this work
AD733	GACTGAATTCTCAGCTAATTAAGCTTTTAAAAGT	this work
AD734	GACGATGATAAGGGTGGAGCAGGTGGATCCGGGGAGTTTCTGG	this work
AD735	GACTGGATCCT*TGACTGA*ATGAGCTCCCCGAAACGCCC	this work
AD736	ACCACCAGAGCCGCCCGACCCACCACCAATATTCAGCTCGTCAT	this work
AD759	GACTAGATCTGGCGCAGGTGTGGATAACAAATTCAATAAAGAAAAGA	this work
AD762	GACTAGATCTGGCGCAGGTAGCTCCCCGAAACGCCCTAA	this work
AD768	GACTGAATTCACCTGCGCCCTTGTACAGCTCGTCCATGC	this work
AD769	GACTGAATTCACCTGCGCCACCAATATTCAGCTCGTCATAGA	this work
AD894	GATCTC**ATG**AAAATATCATCATTTATTTCTACATCACTGC	this work
AD895	GATCGAATTCGCGCAGATCTTCCGCCGGAACCCTGAGGGCTTTCAGTGC	this work
AD903	GACTGTCGACGCAAGCTT*TTA*AAGTTCTTTTG	this work
AD904	GACTGTCGACTCAGCTAATTAAGCTTTTAAAAGT	this work
AD921	GACTAGATCT*TTGACTGA*ATGGGTGGTATCAGTATTTGGC	this work
AD933	GACTTC***ATG****AACTTATCATTAAGCGATCTTCATCGTCAGGTATCTCGATTGGTGCAG*GGAGGTAGATCTGGCGCAGGTGTCTTCACACTCGAAGATTTCGTT	this work
AD934	GACTAAGCTTTTAGAATTCGCCTGCACC*CTTATCATCGTCGTCCTTGTAGTC*ACCTCCCGCCAGAATGCGTTCGCA	this work

Supplementary Table 3: Primers used in this study

Red, restriction sites; blue, linker; purple/bold = start/stop sites.

Supplementary References

1. Zimmerman, S. P. *et al.* Tuning the Binding Affinities and Reversion Kinetics of a Light Inducible Dimer Allows Control of Transmembrane Protein Localization. *Biochemistry* **55**, 5264–71 (2016).
2. Diepold, A., Kudryashev, M., Delalez, N. J., Berry, R. M. & Armitage, J. P. Composition, Formation, and Regulation of the Cytosolic C-ring, a Dynamic Component of the Type III Secretion Injectisome. *PLOS Biol.* **13**, e1002039 (2015).
3. Ittig, S. J. *et al.* A bacterial type III secretion-based protein delivery tool for broad applications in cell biology. *J. Cell Biol.* **211**, 913–31 (2015).
4. Shi, L., Yu, B., Cai, C.-H. & Huang, J.-D. Angiogenic inhibitors delivered by the type III secretion system of tumor-targeting Salmonella typhimurium safely shrink tumors in mice. *AMB Express* **6**, 56 (2016).
5. Walker, B. J., Stan, G.-B. V & Polizzi, K. M. Intracellular delivery of biologic therapeutics by bacterial secretion systems. *Expert Rev. Mol. Med.* **19**, e6 (2017).
6. Shimizu-Sato, S., Huq, E., Tepperman, J. M. & Quail, P. H. A light-switchable gene promoter system. *Nat. Biotechnol.* **20**, 1041–1044 (2002).
7. Reichhart, E., Ingles-Prieto, A., Tichy, A.-M., McKenzie, C. & Janovjak, H. A Phytochrome Sensory Domain Permits Receptor Activation by Red Light. *Angew. Chemie Int. Ed.* **55**, 6339–6342 (2016).
8. Kaberniuk, A. A., Shemetov, A. A. & Verkhusha, V. V. A bacterial phytochrome-based optogenetic system controllable with near-infrared light. *Nat. Methods* **13**, 591–7 (2016).
9. Kudryashev, M. *et al.* In situ structural analysis of the Yersinia enterocolitica injectisome. *Elife* **2**, e00792 (2013).
10. Diepold, A. *et al.* A dynamic and adaptive network of cytosolic interactions governs protein export by the T3SS injectisome. *Nat. Commun.* **8**, 15940 (2017).
11. Kaniga, K., Delor, I. & Cornelis, G. R. A wide-host-range suicide vector for improving reverse genetics in Gram-negative bacteria: inactivation of the blaA gene of Yersinia enterocolitica. *Gene* **109**, 137–41 (1991).
12. Milne-Davies, B. *et al.* Life After Secretion—Yersinia enterocolitica Rapidly Toggles Effector Secretion and Can Resume Cell Division in Response to Changing External Conditions. *Front. Microbiol.* **10**, 2128 (2019).
13. De Leeuw, E., Porcelli, I., Sargent, F., Palmer, T. & Berks, B. C. Membrane interactions and self-association of the TatA and TatB components of the twin-arginine translocation pathway. *FEBS Lett.* **506**, 143–148 (2001).
14. Palmer, T. & Berks, B. C. The twin-arginine translocation (Tat) protein export pathway. *Nat. Rev. Microbiol.* **10**, 483–496 (2012).
15. Kawano, F., Suzuki, H., Furuya, A. & Sato, M. Engineered pairs of distinct photoswitches for optogenetic control of cellular proteins. *Nat. Commun.* **6**, 6256 (2015).
